# The Postsynaptic Density: There Is More than Meets the Eye

**DOI:** 10.3389/fnsyn.2016.00023

**Published:** 2016-08-19

**Authors:** Ayse Dosemeci, Richard J. Weinberg, Thomas S. Reese, Jung-Hwa Tao-Cheng

**Affiliations:** ^1^Laboratory of Neurobiology, National Institute of Neurological Disorders and Stroke (NINDS), National Institutes of Health (NIH)Bethesda, MD, USA; ^2^Department of Cell Biology and Physiology, University of North Carolina, Chapel HillChapel Hill, NC, USA; ^3^Electron Microscopy Facility, National Institute of Neurological Disorders and Stroke (NINDS), National Institutes of Health (NIH)Bethesda, MD, USA

**Keywords:** postsynaptic density, PSD, pallium, electron microscopy, EM, Shank, Homer, CaMKII

## Abstract

The postsynaptic density (PSD), apparent in electron micrographs as a dense lamina just beneath the postsynaptic membrane, includes a deeper layer, the “pallium”, containing a scaffold of Shank and Homer proteins. Though poorly defined in traditionally prepared thin-section electron micrographs, the pallium becomes denser and more conspicuous during intense synaptic activity, due to the reversible addition of CaMKII and other proteins. In this Perspective article, we review the significance of CaMKII-mediated recruitment of proteins to the pallium with respect to both the trafficking of receptors and the remodeling of spine shape that follow synaptic stimulation. We suggest that the level and duration of CaMKII translocation and activation in the pallium will shape activity-induced changes in the spine.

Excitatory glutamatergic synapses examined with the electron microscope typically display a pronounced postsynaptic density (PSD), which appears in conventional electron micrographs as an approximately 30 nm thick electron-dense structure applied to the postsynaptic membrane (Palay, 1956). The large majority of excitatory synapses in the vertebrate CNS release glutamate as neurotransmitter. Ionotropic glutamate receptors concentrate at the PSD, where specialized molecules anchor them and regulate their trafficking; modulation of their expression and trafficking plays a key role in synaptic plasticity. Decades of research show that much of the story of this modulation resides in the PSD.

Using special stains, Valtschanoff and Weinberg (2001) uncovered a distinct layer ~50 nm thick immediately adjacent and deep to the PSD. Though previously reported, this “subsynaptic web” (DeRobertis, 1964) had been largely ignored for decades, because it is difficult to distinguish from the rest of the spine cytoplasm after standard histological procedures. Extending this work, Petralia et al. (2005) reported high levels of immunoEM label for both Shank and Homer in the same region, which was referred to as the “subjacent” area.

The subsynaptic web becomes more prominent after short bouts of stimulation, reflecting the reversible incorporation of additional proteins. Because this part of the PSD comprises different proteins and displays a more labile organization than the “core” layer lining the synaptic membrane, it merits a distinct name; we propose to designate this separate structural and functional layer of the PSD as its *pallium* or mantle (Figure 1). Here we present a perspective on PSD structure that highlights potential functions carried out in the pallium.

**Figure 1:**
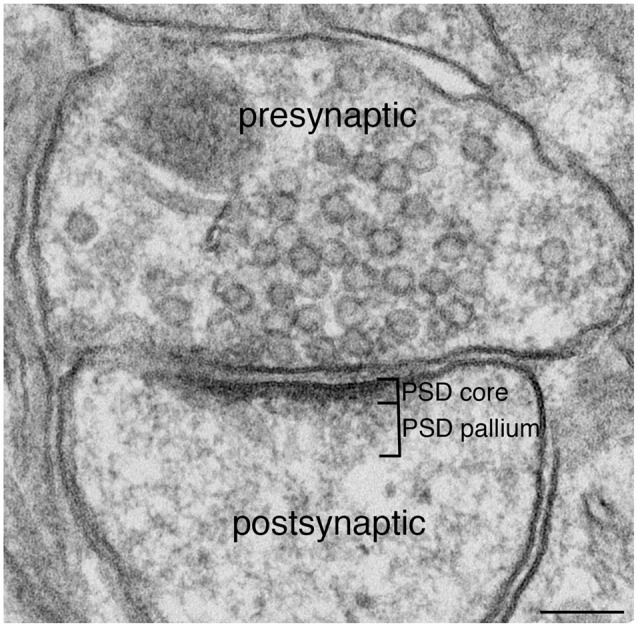
**Excitatory synapse.** Excitatory glutamatergic synapses typically display a prominent electron-dense zone juxtaposed to the postsynaptic membrane, traditionally known as the postsynaptic density (PSD). Extending deeper from the PSD core is a specialized region we call the PSD pallium. Scale bar: 0.1 μm.

## Core Layer of the PSD

Though highly variable, PSDs in the rodent forebrain contain on average 300–400 copies of PSD-95 and related membrane-associated guanylate kinase (MAGUK) molecules (Chen et al., 2005; Sugiyama et al., 2005). PSD-95 is localized in the core layer of the PSD. Comparing immunogold labeling with antibodies against its N- and C-termini shows that PSD-95 is oriented with its N-terminus near the plane of the postsynaptic membrane and its C-terminus deep in the spine (Chen et al., 2008). Electron microscopic tomography reveals vertical filaments extending from the postsynaptic membrane in numbers and dimensions expected for PSD-95 (Chen et al., 2008), and these filaments are depleted by acute knockdown of PSD-95 (Chen et al., 2011). Thus, PSD-95-containing filaments oriented vertically to the postsynaptic membrane represent a major structural component of the core layer (Chen et al., 2008). This close apposition to the membrane puts PSD-95 in a position to bind neurotransmitter receptors. Indeed, there is substantial evidence indicating the association of PSD-95 with both NMDA and AMPA types of glutamate receptors.

EM tomography also reveals filaments of different lengths, oriented parallel to the postsynaptic membrane, that appear to contact the deep (C-terminal) ends of PSD-95 filaments (Chen et al., 2008). The dimensions of one frequently-encountered type of horizontal filament corresponds to that expected for guanylate kinase-associated protein (GKAP), consistent with biochemical work demonstrating that GKAP can bind PSD-95 near its C-terminal (Kim et al., 1997). GKAP is in the PSD at a ratio of approximately one GKAP for two PSD-95s (Lowenthal et al., 2015) so a layer of GKAP cross-linked with PSD-95 could provide an interface between the core layer and the pallium of the PSD. Like PSD-95 (Yang et al., 2011), GKAP stays put during brief stimulation (Tao-Cheng et al., 2015). Thus, the dense matrix of PSD-95/MAGUK filaments capped by GKAPs provides a stable platform for the subjacent pallium.

## Pallial Layer of the PSD

Early biochemical analyses indicated that the PSD complex contains several types of scaffolding molecules in addition to MAGUKs and GKAPs (Box 1). Two-hybrid and co-immunoprecipitation experiments led to the identification of the Shank/ProSAP family of proteins, which bind to GKAPs (Boeckers et al., 1999a,b; Naisbitt et al., 1999). Similar two-hybrid studies identified the Homer family of proteins as binding partners for Shanks and the two types of proteins were shown to co-immunoprecipitate from brain extracts (Tu et al., 1999). Both Shank and Homer family proteins are highly enriched in PSD fractions (Xiao et al., 1998; Naisbitt et al., 1999) and co-purify as large complexes, with PSD-95, GKAP and other PSD constituents (Collins et al., 2006; Dosemeci et al., 2007).

Box 1 **Major scaffold proteins in the PSD.**

Names used in the present article are listed along with synonyms.

**MAGUKs** (membrane-associated guanylate kinases).

The most abundant MAGUK at the mammalian PSD is **PSD-95** (DLG4, SAP90); others include SAP97 (DLG1), PSD-93 (DLG2, Chapsyn-110), and SAP102 (DLG3).

**GKAP** (guanylate kinase-associated protein) also called SAPAP (SAP90/PSD-95-associated protein), or disks large-associated protein.

**Shank** (SH3 and multiple ankyrin repeat domains protein) also called ProSAP (Proline-rich synapse-associated protein), Synamon and CortBP (Cortactin binding protein).

**Homer** also called Vesl, Cupidin, and PSD-Zip45.

**Figure 2:**
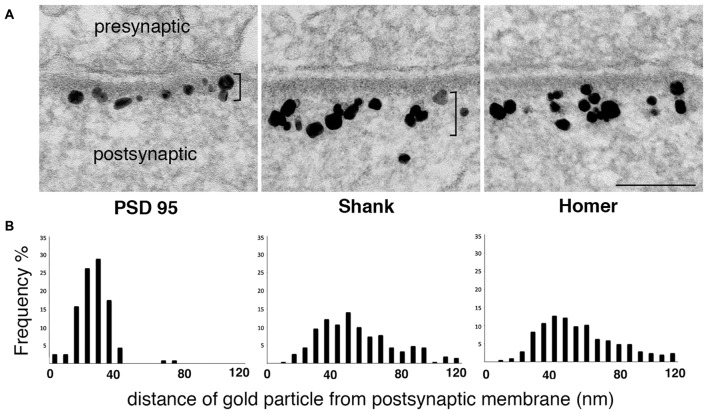
**Label for two PSD scaffold proteins, Shank and Homer, lies mostly outside the PSD core. (A)** Electron micrographs of asymmetric synapses in cultured hippocampal neurons immunogold labeled with antibodies for three major PSD scaffold proteins. Silver-enhanced gold label appears as black particles of heterogeneous size (EM in middle panel from Tao-Cheng et al., 2015). The PSD core with PSD-95 label and the PSD pallium with Shank and Homer labels are marked by brackets on the left and middle panels respectively. Scale bar: 0.1 μm. **(B)** Frequency histograms depicting the distribution of label at the postsynaptic compartment in a typical experiment. While label for PSD-95 is within the electron-dense material, label for Shank and Homer is concentrated in a deeper region we designate as the *pallium* of the PSD.

While biochemical studies identify Shank and Homer as part of the PSD complex, immunoEM studies show that label for these proteins concentrates outside the electron-dense material conventionally defined as the PSD (Figure 2). Although the reported distributions of label for these proteins differ somewhat between groups, immunoEM studies typically find that much of the postsynaptic immunogold label for both Shank and Homer lie in a layer immediately below the PSD core (Tu et al., 1999; Valtschanoff and Weinberg, 2001; Petralia et al., 2005; Rostaing et al., 2006; Tao-Cheng et al., 2010, 2014a).

These data imply that a network containing Shanks and Homer extends the PSD scaffold well beyond the classically recognized electron-dense material. This second layer, which we term the *pallium*, is likely pegged to the PSD core through GKAPs, which can simultaneously associate with PSD-95 and Shanks. *In vitro* studies report that Shanks can associate with each other through their SAM domains to form sheet-like structures (Baron et al., 2006) and Homers can polymerize into tetramers that can cross-link Shanks (Hayashi et al., 2009). Furthermore, purified C-terminal Shank and Homer form a mesh-like matrix when mixed together (Hayashi et al., 2009). Thus, Shank and Homer proteins in the pallium are likely to form an extended scaffold. The side of this scaffold facing the cleft is continuous with the PSD core, whereas the cytoplasmic side, characterized by an extremely rough surface (Petersen et al., 2003) merges imperceptibly into the central zone of the spine head.

## Activity-Induced Changes at the Pallium

### The Pallium Becomes More Electron-Dense as CaMKII Accumulates

**Figure 3:**
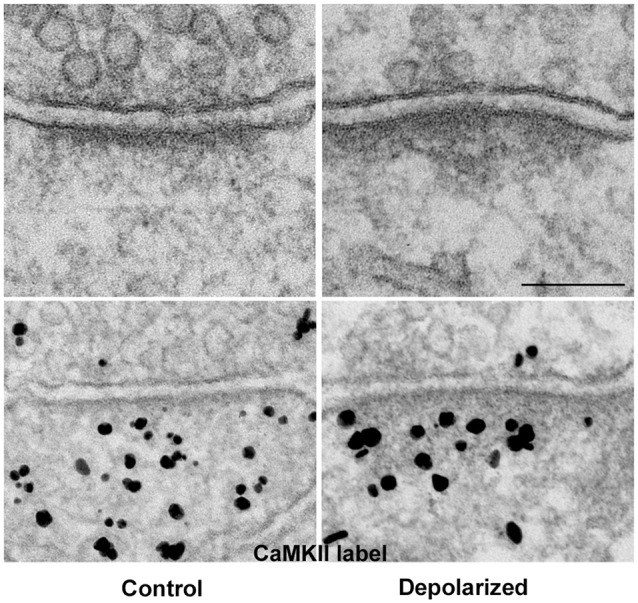
**The PSD pallium becomes electron-dense as CaMKII and other proteins are recruited under excitatory conditions.** Electron micrographs of asymmetric synapses in cultured hippocampal neurons under basal conditions (left) and after depolarization in medium containing high K^+^ (right). Upon intense synaptic activity the pallium of the PSD becomes electron-dense due to the accumulation of proteins, including CaMKII (bottom panels). Scale bar: 0.1 μm.

Thin-section EM of cultured hippocampal neurons reveals increased electron density at the pallium under excitatory conditions (Figure 3: top panels), a phenomenon we have described as PSD “thickening” (Dosemeci et al., 2001). The increase in electron density likely reflects the addition of proteins, and immunoEM shows significant increase in CaMKII immunolabel within the PSD complex under excitatory conditions (Figure 3: bottom panels, Dosemeci et al., 2001). Similar morphological changes at the PSD, accompanied by the accumulation of CaMKII and other proteins, also occur under the excitotoxic conditions promoted by ischemia (Suzuki et al., 1994; Hu et al., 1998; Martone et al., 1999). The duration of the morphological and compositional changes at the PSD appears to vary according to the type of stimulation; importantly, more sustained modification is observed following an LTP-inducing protocol (Otmakhov et al., 2004).

#### Role of CaMKII as a Kinase

CaMKII, a Ca^2+^-regulated serine-threonine protein kinase, is present at very high concentrations in neurons, with a relative abundance reaching ~1–2% of total protein in the cerebral cortex and hippocampus (Erondu and Kennedy, 1985). Within neurons, CaMKII is especially prominent in the PSD pallium (Petralia et al., 2005; Ding et al., 2013). Activation of CaMKII plays a pivotal role in certain types of synaptic modification, most notably NMDA-dependent LTP (reviews: Lisman et al., 2012; Shonesy et al., 2014).

The CaMKII holoenzyme is a dodecamer made of individually active subunits, with α- and β-isoforms prevalent in brain. Subunits within a single holoenzyme can phosphorylate one another, a process called autophosphorylation. Upon autophosphorylation in the presence of Ca^2+^, CaMKII becomes autonomous, allowing enzymatic activity to persist beyond the duration of Ca^2+^ elevation (reviews: Hell, 2014; Shonesy et al., 2014). Autonomous CaMKII can remain at the PSD pallium after the cessation of excitatory stimuli (Dosemeci et al., 2002; Otmakhov et al., 2004). Continued co-localization of autonomous CaMKII with its substrates at the pallium would counteract dephosphorylation by phosphatases and thus help maintain phosphorylation of CaMKII substrates.

The accumulation and activation of CaMKII at the pallium triggers further changes at the PSD. Some of these have been studied in depth, such as the incorporation of AMPA receptors into the postsynaptic membrane (review: Lisman et al., 2012). ImmunoEM studies also reveal activity-induced increases in the levels of a number of proteins at the pallium, including SynGAP, AIDA-1 and Shanks, in parallel with CaMKII. Importantly, the redistribution of all these PSD components is blocked by the application of a specific CaMKII inhibitor (Yang et al., 2013; Tao-Cheng et al., 2014b; Dosemeci et al., 2016), indicating that the activation of CaMKII is required for the redistribution of these PSD components.

#### Role of CaMKII as a Dynamic Structural Element

In addition to its function as a protein kinase, accumulating evidence suggests that CaMKII in the pallium may act as a scaffold. Bingol et al. (2010) show that activity-induced translocation of autophosphorylated α-CaMKII, but not its kinase activity, is responsible for the translocation of proteasomes into synapses. Similarly, activity-induced CaMKII translocation is responsible for parallel accumulation of the deubiquitinase CYLD, specific for K63-linked polyubiquitins, to the PSD (Thein et al., 2014). Since K63-linked polyubiquitination inhibits interaction of proteins with proteasomes (Nathan et al., 2013) CYLD activity is expected to facilitate protein binding to proteasomes. Thus, CaMKII accumulated at the pallium appears to constitute a structural platform that brings together components that may work synergistically to promote local degradation of proteins.

Activity-induced translocation of CaMKII also may mediate modifications in spine morphology. CaMKII holoenzymes at the spine cytoplasm serve as actin bundling elements (Okamoto et al., 2007) and dissociation of CaMKII from F-actin upon autophosphorylation destabilizes the actin cytoskeleton (Kim et al., 2015). Thus the translocation of CaMKII from the actin network to the PSD pallium can trigger synchronized changes at the PSD and actin cytoskeleton.

### SynGAP Levels Decrease in the PSD Core and Increase in the Pallium

SynGAP is an enzymatic regulator of Ras, a small GTPase. However, as one of the most abundant proteins at the PSD (at levels exceeding even those of PSD-95, its binding partner at the PSD core), SynGAP is likely to also play a non-enzymatic role. ImmunoEM shows that SynGAP label is concentrated at the PSD core (Yang et al., 2011, 2013). After strong depolarization, labeling for SynGAP significantly decreases at the PSD core while increasing at the pallium (Yang et al., 2011, 2013). Under the same conditions, label for PSD-95 does not change its localization at the PSD (Yang et al., 2011), implying dissociation of the two molecules. Further studies indicate that the release of SynGAP from PSD-95 and its exit from the PSD core require CaMKII-mediated phosphorylation (Yang et al., 2013; Araki et al., 2015).

NMDA- or high K^+^-induced changes in the distribution of SynGAP are reversed within 30 min after cessation of the excitatory conditions. In contrast, a stimulation protocol (glycine in the absence of Mg^2+^) that leads to sustained increases in synaptic efficacy (chem-LTP) also leads to a sustained exclusion of SynGAP from the spine (Araki et al., 2015) indicating a correlation between LTP and the removal of SynGAP. It appears likely that a wide range of excitatory stimuli promotes translocation of SynGAP out of the PSD core, but that only under LTP-promoting conditions is the molecule removed on a long-term basis. The extent and maintenance of SynGAP phosphorylation at the pallium may determine its subsequent movement. In this regard, it is interesting that SynGAP is most efficiently phosphorylated by the autonomous form of CaMKII in the absence of Ca^2+^ (Dosemeci and Jaffe, 2010).

What might be the functional implications of activity-induced redistribution of SynGAP at the PSD? Both SynGAP and members of the transmembrane AMPA receptor regulatory protein family (TARPs) can bind to the same PDZ domains on PSD-95 (Kim et al., 1998; Schnell et al., 2002; Dakoji et al., 2003) and thus may compete for association with PSD-95. Considering its high abundance in the PSD, SynGAP is likely to block TARP binding to PDZ domains on PSD-95 under basal conditions. Thus, the removal of SynGAP during activity may be a prerequisite for the anchoring of AMPA receptors to the PSD, explaining the well-documented role of SynGAP as an inhibitor of AMPA receptor insertion (Rumbaugh et al., 2006). This function may account for the remarkable abundance of SynGAP at the PSD.

### AIDA-1 Levels Decrease in the PSD Core and Increase in the Pallium

Quantitative mass spectrometric analysis of PSD fractions reveals that Amyloid-β protein precursor Intracellular Domain-Associated 1 (AIDA-1, also known as EB-1 and ANKS1B) is a major component of the PSD complex, with a PSD-95/AIDA-1/GKAP stoichiometry of 2:1:1 (Lowenthal et al., 2015). Like SynGAP, AIDA-1 binds directly to PSD-95 at the same domains that bind TARPs (Jordan et al., 2007) and therefore could also interfere with the anchoring of AMPA receptors. By immunoEM, the AIDA-1 label is mostly located within the PSD core at rest (Jacob et al., 2010; Dosemeci et al., 2015). Under excitatory conditions AIDA-1 label at the PSD core is significantly reduced with a concomitant increase at the pallium (Dosemeci et al., 2015). The reversible CaMKII-mediated redistribution of AIDA-1 at the PSD under excitatory conditions (Dosemeci et al., 2016) parallels that of SynGAP. We speculate that different regulatory mechanisms may trigger dissociation of SynGAP and AIDA-1 from PSD-95.

### Shank Levels Increase in the Pallium

Shanks, a protein scaffold family concentrated in the PSD, are encoded by three genes, *Shank1*, *Shank2* and *Shank3*, each giving rise to multiple splice variants. Shank mutations have been linked to autism and other neurodevelopmental/neuropsychiatric disorders (reviews: Grabrucker et al., 2011; Sala et al., 2015). All Shank isoforms have a similar molecular organization, with specialized domains that can associate with GKAPs, Homers, and other Shanks. Analysis of immunoEM data suggests that Shanks at the PSD are organized into a proximal pool, close enough to the interface between the core and pallium to associate with GKAP, and a distal (deeper) pool that may be stabilized through association with Homers and/or with other Shanks. Under excitatory conditions, Shanks accumulate preferentially in the distal pool (Tao-Cheng et al., 2015).

Shanks promote maturation of dendritic spines and the enlargement of spine heads (Sala et al., 2001), although the precise mechanism remains unclear. Considering that changes in spine shape and size involve reorganization of the actin cytoskeleton, it is likely that Shanks regulate spine morphology through interaction with actin. Indeed, Shanks associate with three actin-regulating proteins, Insulin Receptor Substrate Protein 53 (IRSp53), Abp1 and cortactin.

The actin binding protein IRSp53 (also called BAIAP2; see review by Kang et al., 2016) is a major PSD component, with a molar ratio of IRSp53 to PSD-95 of 1:4 (Lowenthal et al., 2015). IRSp53 is located between layers containing Shank and PSD-95, relatively close to the postsynaptic membrane (Burette et al., 2014), where it may function as a linker between the actin cytoskeleton and the plasma membrane (Scita et al., 2008; Burette et al., 2014). Indeed, IRSp53 contains an actin-binding BAR-like domain that can induce changes in membrane curvature (Zhao et al., 2011) and thus may be involved in activity-induced changes in the curvature of the synapse (Burette et al., 2014).

Abp1, which can associate simultaneously with actin and Shanks, may link the actin cytoskeleton to the PSD (Qualmann et al., 2004). Abp1 preferentially interacts with dynamic rather than static F-actin structures (Kessels et al., 2000) and more Abp1 becomes incorporated into Shank-positive synapses during activity (Qualmann et al., 2004). Overexpression of Abp1 increases the density of mushroom-shaped spines; importantly, its association with Shank is necessary for Abp1-mediated regulation of spine morphology (Haeckel et al., 2008).

Another protein that associates with both Shanks and actin is cortactin, although immunoEM shows that cortactin concentrates mainly at the central region of the spine (Racz and Weinberg, 2004). In contrast to Abp1, cortactin exits the spines during activity (Hering and Sheng, 2003). While Abp1 overexpression increases mushroom spine density (Haeckel et al., 2008), cortactin overexpression causes elongation of spines (Hering and Sheng, 2003).

The above considerations lead us to suggest a model for activity-induced modification of spine morphology: under basal conditions, cytosolic Shanks within the spine are pegged to the actin cytoskeleton through cortactin and held outside of the pallium. During activity, CaMKII-mediated phosphorylation releases Shanks from cortactin. The Shank thus released accumulates at the pallium, while cortactin exits the spine. Shank accumulated at the pallium could then recruit Abp1 to promote remodeling of the actin cytoskeleton around the pallium.

## Actin and the PSD

There are several reports that filaments of F-actin, the primary cytoskeletal component of the spine, contact the PSD (Gulley and Reese, 1981; Landis and Reese, 1983; Fifková, 1985), especially at its periphery (Burette et al., 2012), though these contacts are likely highly variable, considering the dynamic nature of F-actin. While the molecular basis of the attachment of actin filaments to the PSD remains uncertain, a number of PSD molecules are plausible candidates. As discussed above, two Shank binding proteins, IRSp53 and Abp1, may provide a link between the PSD and the actin cytoskeleton.

Yet another point of interaction of actin with the PSD may be through the ubiquitous actin binding protein α-actinin. *In vitro* assays show that α-actinin can form a ternary complex with the PSD proteins, densin and CaMKII (Walikonis et al., 2001). Loss of α-actinin-2 in rat hippocampal neurons creates an increased density of immature, filopodia-like protrusions that fail to mature into a mushroom-shaped spine during development (Hodges et al., 2014).

## Conclusion

The pallium is an extension of the electron-dense PSD core, demarcated by strong immunolabeling for two PSD scaffold proteins, Shank and Homer. This specialized zone, sandwiched between the electron-dense PSD core and the actin “spinoskeleton”, is highly dynamic, exhibiting striking changes during synaptic activity. Under conditions of strong excitation, the pallium becomes electron-dense, with the addition of CaMKII and several other proteins (Table 1). Activation and/or translocation of CaMKII is necessary for the recruitment of other components to the pallium.

**Table 1:** **Redistribution of postsynaptic density (PSD) components during activity**.

	CaMKII	SynGAP	AIDA-1	Shank	CYLD
Core		decrease	decrease		
Pallium	increase	increase	increase	increase	increase

*Changes observed in immunogold label density for selected proteins following treatment of cultured hippocampal neurons with media containing NMDA or high K^+^*.

The pallium can be viewed as a hub where several proteins converge during activity. Accumulating evidence on the movements of individual proteins suggests a mechanism for concerted insertion of receptors to the PSD and re-organization of the actin spinoskeleton, both mediated by CaMKII. Upon synaptic stimulation, translocation and activation of CaMKII cause SynGAP and AIDA-1 to move out of the PSD core and accumulate at the pallium (Yang et al., 2013; Dosemeci et al., 2015, 2016). We propose that the movement of SynGAP (and perhaps also of AIDA-1) vacates “slots” on PSD-95, providing a window of opportunity for the insertion of AMPA receptors. Simultaneous CaMKII-mediated accumulation of Shanks at the pallium (Tao-Cheng et al., 2014b), on the other hand, may enable actin reorganization around the PSD. CaMKII also acts as a dynamic structural element whose activity-induced translocation changes the molecular organization within the spine. Dissociation of CaMKII from F-actin causes destabilization and reorganization of the actin cytoskeleton (Kim et al., 2015). Subsequent accumulation of CaMKII at the pallium docks elements that regulate protein turnover (Bingol et al., 2010; Thein et al., 2014).

In summary, activation of CaMKII and its translocation to the pallium under excitatory conditions trigger a chain of events poised to elicit profound effects on the organization of the PSD complex and of the actin cytoskeleton that could synchronize receptor trafficking with changes in spine morphology. We speculate that the degree and duration of CaMKII accumulation and activity at the pallium, promoted by different types of excitatory stimuli, may determine the type and level of synaptic modification.

## Author Contributions

All authors contributed to the writing of the manuscript.

## Conflict of Interest Statement

The authors declare that the research was conducted in the absence of any commercial or financial relationships that could be construed as a potential conflict of interest.
